# Screening for Small Molecule Inhibitors of Statin-Induced APP C-terminal Toxic Fragment Production

**DOI:** 10.3389/fphar.2017.00046

**Published:** 2017-02-15

**Authors:** Karen S. Poksay, Douglas J. Sheffler, Patricia Spilman, Jesus Campagna, Barbara Jagodzinska, Olivier Descamps, Olivia Gorostiza, Alex Matalis, Michael Mullenix, Dale E. Bredesen, Nicholas D. P. Cosford, Varghese John

**Affiliations:** ^1^Bredesen Lab, Buck Institute for Research on Aging, NovatoCA, USA; ^2^Cancer Metabolism and Signaling Networks Program, Sanford Burnham Prebys Medical Discovery Institute, La JollaCA, USA; ^3^Drug Discovery Lab, Department of Neurology, University of California, Los AngelesCA, USA; ^4^Enzo Life Sciences, Inc., FarmingdaleNY, USA

**Keywords:** AD – Alzheimer’s disease, APP – amyloid precursor protein, APP-C31 –C-terminal 31 amino acid fragment of APP, APPΔC31 – N-terminal APP fragment minus C-terminal 31 amino acids (alphabetical), statins

## Abstract

Alzheimer’s disease (AD) is characterized by neuronal and synaptic loss. One process that could contribute to this loss is the intracellular caspase cleavage of the amyloid precursor protein (APP) resulting in release of the toxic C-terminal 31-amino acid peptide APP-C31 along with the production of APPΔC31, full-length APP minus the C-terminal 31 amino acids. We previously found that a mutation in APP that prevents this caspase cleavage ameliorated synaptic loss and cognitive impairment in a murine AD model. Thus, inhibition of this cleavage is a reasonable target for new therapeutic development. In order to identify small molecules that inhibit the generation of APP-C31, we first used an APPΔC31 cleavage site-specific antibody to develop an AlphaLISA to screen several chemical compound libraries for the level of N-terminal fragment production. This antibody was also used to develop an ELISA for validation studies. In both high throughput screening (HTS) and validation testing, the ability of compounds to inhibit simvastatin- (HTS) or cerivastatin- (validation studies) induced caspase cleavage at the APP-D720 cleavage site was determined in Chinese hamster ovary (CHO) cells stably transfected with wildtype (wt) human APP (CHO-7W). Several compounds, as well as control pan-caspase inhibitor Q-VD-OPh, inhibited APPΔC31 production (measured fragment) and rescued cell death in a dose-dependent manner. The effective compounds fell into several classes including SERCA inhibitors, inhibitors of Wnt signaling, and calcium channel antagonists. Further studies are underway to evaluate the efficacy of lead compounds – identified here using cells and tissues expressing wt human APP – in mouse models of AD expressing mutated human APP, as well as to identify additional compounds and determine the mechanisms by which they exert their effects.

## Introduction

Alzheimer’s disease (AD) is a progressive neurodegenerative disease characterized by cognitive and functional decline. Pathologically, it is characterized by two types of lesions in the brain: extracellular senile plaques consisting primarily of amyloid precursor protein (APP)-derived amyloid-β (Aβ) peptide (Ikeda et al., 1987; Hardy and Allsop, 1991) and intracellular neurofibrillary tangles consisting largely of hyper-phosphorylated microtubule-associated tau protein (Goedert, 1993; Dickson, 1997). Synapse loss and neuronal cell death represent the basis for cognitive impairment in AD (Scheff et al., 1996; Lacor et al., 2007), and the C-terminal caspase cleavage of APP resulting in release of a 31 amino acid C-terminal fragment (APP-C31) has been shown to be a likely contributor to neuronal death in AD (Lu et al., 2000; Banwait et al., 2008).

**FIGURE 1:**
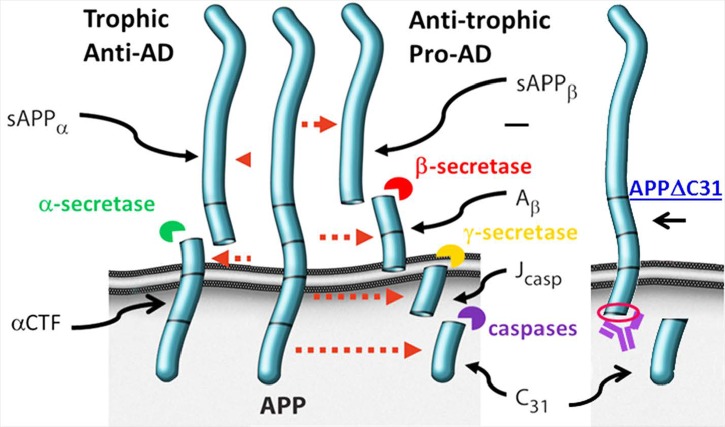
**Amyloid precursor protein (APP) proteolytic processing.** Full-length amyloid precursor protein (FL APP) may be cleaved by α-secretase (putatively ADAM10) to produce trophic fragment soluble APPalpha (sAPPα) and the alpha C-terminal fragment (α-CTF). Alternatively, FL APP may be cleaved by β-secretase (BACE1), generating sAPPβ and β-CTF; the latter is cleaved by γ-secretase, producing amyloid-beta (Aβ) and the APP intracellular domain (AICD). Should AICD undergo caspase cleavage, Jcasp and C31 are generated. FL APP can also undergo caspase cleavage, producing APPΔC31 and the C31 fragment. APPΔ31 is the fragment that is detected by the assays described here. The antibody generated by Enzo was raised against the C-terminal amino acids comprising the “neo” epitope (circled in red) of APPΔ31 after caspase cleavage of APP.

As shown in **Figure 1**, APP may be cleaved by an α-secretase (putatively ADAM10) to generate soluble APPα (sAPPα) and the alpha C-terminal fragment (αCTF) which support synaptic connections and neuronal survival. Alternatively, APP may be cleaved by β-secretase BACE1 resulting in sAPPβ and βCTF, the latter of which can then be further cleaved by the γ-secretase complex to give Aβ of a variety of lengths and the APP intracellular domain (AICD). AICD (or larger APP species) can undergo caspase cleavage giving rise to, for example, Jcasp and APP-C31. Full-length APP is also subject to caspase cleavage, resulting in generation of APPΔC31 (measured here) and APP-C31. APPΔC31 has been found in higher levels in the brains of patients with AD as compared to controls, as determined by immunoblotting (Lu et al., 2000) and immunohistochemistry (Banwait et al., 2008) using a caspase cleavage-site specific antibody. This C-terminal APP proteolytic cleavage is markedly increased in early stages of AD as related to Braak staging (Braak and Braak, 1997); increased staining is seen in the hippocampal region and around some plaque and tangle-like structures as well as in peri- and intra- neuronal regions of AD brains, whereas minimal reactivity is seen in age-matched control patients (Banwait et al., 2008).

The original finding that caspase cleavage of APP and generation of APP-C31 was toxic and could lead to apoptosis *in vitro* was presented in Lu et al. (2000), and additional studies showed production of APP-C31 could be mediated by Aβ interaction with full-length APP, resulting in dimerization and caspase cleavage (Shaked et al., 2006). Studies by McPhie et al. (2001) demonstrated that overexpression of the APP-C31 peptide in primary cortical neurons via an HSV vector significantly increased apoptosis over controls, that the generation of APP-C31 occurred in the absence of γ-secretase cleavage, and that this toxic pathway was enhanced in the presence of familial Alzheimer’s disease (FAD) β-site mutations.

In earlier studies to determine the impact of inhibition of APP-C31 production *in vivo*, we created transgenic mice similar to the well-characterized J9 and J20 PDAPP models of AD described elsewhere (Hsia et al., 1999; Mucke et al., 2000), but with a D664A mutation that prevented caspase cleavage and APP-C31 generation (Galvan et al., 2006). J9 and J20 mice express human APP (hAPP) containing the Swedish and Indiana FAD mutations under the control of the platelet-derived growth factor (PDGF) promoter, with expression being higher in J20. The Swedish mutation increases BACE cleavage of APP and the Indiana mutation increases Aβ1-42 production over other Aβ species. In our B21 and B254 lines with the D664A mutation having expression levels similar to the J9 and J20 lines, respectively, we found that much of the AD phenotype was ameliorated including changes in long-term potentiation (LTP) and loss of hippocampal volume (Galvan et al., 2006). In another study (Harris et al., 2010), comparing J20 mice to B254 mice with the D664A mutation, the B254 mice did not show the dramatic behavioral improvements seen in the original study, however in that study B254 mice were found to have significantly higher Aβ production and plaque load compared to the J20 mice, therefore it was stated by the authors of this study that the D664A mutation may have had a protective effect after all, making hAPP-B254 mice relatively resistant against higher levels of Aβ. Thus, induction of APP-C31 production may be one way in which Aβ exerts its deleterious effects, and thus inhibition of this cleavage could be a new target for AD drug discovery.

In this work, we set out to identify small molecule inhibitors of the APP-C31 generating cleavage pathway. Such an effort, we posited, could lead to chemical-genetic tools that could reveal mechanisms and pathways in APP-C31 production and neurotoxicity. For our studies, we measured APPΔC31 rather than APP-C31 levels as the latter peptide is short-lived and difficult to detect. Our prior studies have shown the ability of statins, particularly lipophilic statins such as simvastatin and cerivastatin, to stimulate APPΔC31 production (Descamps et al., 2011). As a result, we utilized statins to stimulate the intracellular caspase cleavage of APP at D720 (APP_751_ numbering) *in vitro* in CHO-7W cells stably overexpressing hAPPwt so as to be able to more easily detect and then inhibit this cleavage. The approach described herein, as well as the target of screening – lowering of APPΔC31 and resulting APP-C31 – represents a new approach to therapeutic development in AD that may alter the course of disease in its early stages.

## Materials and Methods

### Anti-APPΔC31 Polyclonal Antibody and Validation

In order to more accurately and sensitively quantify caspase-cleaved APP in cell and tissue lysates, we partnered with Enzo Life Sciences to develop both a cleavage site-specific (neo epitope) polyclonal antibody that recognizes the C-terminus of APPΔC31 resulting from the caspase cleavage and an ELISA (ENZ-ABS445-0100, ADI-900-227, respectively). The immunizing antigen comprised a short peptide sequence on the C-terminus of APPΔC31 (red circle, **Figure 1**).

**FIGURE 2:**
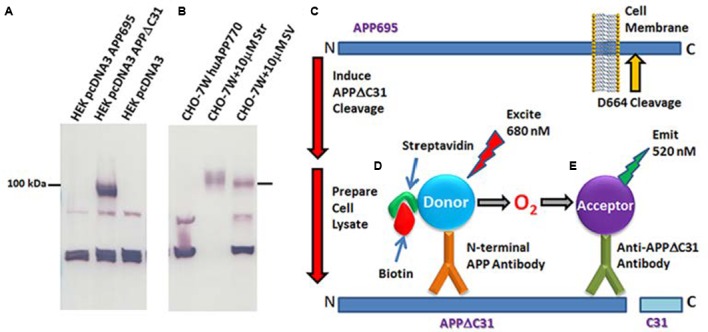
**Anti-APPΔC31 antibody and AlphaLISA.** **(A)** The anti-APPΔC31 antibody only detects APPΔC31 in *pcDNA3-APPΔC31*-transfected (lane 2) human embryonic kidney (HEK 293T) cells, and not in cells transfected with full-length *pcDNA3-APP_695_* (lane one) or empty vector (lane 3). **(B)** Similarly, the antibody detects only APPΔC31 in Chinese hamster ovary cells stably transfected with human *APP_751_* (CHO-7W) when the cells are treated with caspase cleavage-inducing staurosporine (Str) or simvastatin (SV) (both at 10 μM). There is some non-specific labeling of lower MW bands that is seen in both cell types. The AlphaLISA, however, uses a second APP- specific antibody to limit detection to capase-cleaved APP only. **(C)** The caspase cleavage site of APP and its juxtaposition to the plasma membrane is shown (yellow arrow). **(D)** APPΔC31 is bound by both an anti-APP N-terminal specific antibody (bound to donor beads). **(E)** The anti- APPΔC31 antibody (bound to acceptor beads) to allow specific detection of APPΔC31. Only when the donor and acceptor beads are in close proximity to each other does excitation at 680 nm producing O2 result in emission from the acceptor bead at 580 nm that is detectable by the plate reader.

The specificity of the antibody for the neo epitope, rather than full length APP, was validated by immunoblot (**Figures 2A,B**, **Supplementary Figure S1A**) and ELISA (**Supplementary Figure S1B**). Human embryonic kidney (HEK 293T) cells were grown in high glucose DMEM with 10% heat-inactivated fetal bovine serum and 1X antibiotic/antimycotic and transfected with full-length *pcDNA3-APP_695_. pcDNA3-APPΔC31*, or empty pcDNA3 vector using Lipofectamine 2000 (Life Technologies). Two days later, RIPA buffer lysates complemented with complete protease inhibitors (PI, Roche) were prepared. Samples were run on 12% Bio-Rad Criterion gels, transferred onto 0.2 μm PVDF membrane and probed with the anti-APPΔC31 antibody. Anti-rabbit secondary antibody conjugated to HRP (horseradish peroxidase, Amersham) was then incubated with the membrane, signal was generated using chemiluminescence (Thermo) and film exposed. In addition, samples were analyzed by APPΔC31 ELISA.

Similarly, Chinese hamster ovary cells stably transfected with human wildtype (wt) *APP_751_* (CHO- 7W, a kind gift from Dr. Edward Koo) were cultured in DMEM, 10% FBS, 1X antibiotic/antimycotic. They were treated with activated simvastatin or staurosporine both at 10 μM for 24 h. RIPA lysates were prepared using complete protease inhibitors (Roche).

For immunoprecipitation, approximately 300 μg cell lysate supernatants were brought to 250 μL with cold RIPA + PI buffer in microfuge tubes. One μL of 6E10 APP antibody (Covance) was added and the mixture was rotated overnight in a cold room. The next day, 25 μL of protein A/G beads (Santa Cruz Biotech) were added and the samples were rotated for 1.5 h in the cold room. The beads were then centrifuged down and washed four times with cold PBS. Forty μL 1x LDS sample buffer + DTT was added to the pellets, which were heated to 70°C for 10 min, vortexed and centrifuged. Supernatants were loaded onto Nupage 4–12% Bis-Tris gels (Life Technologies) for electrophoresis and were then transferred to 0.2 μm PVDF membrane for immunoblotting with the anti-APPΔC31 antibody.

### AlphaLISA to Detect APPΔC31

A custom AlphaLISA (Perkin-Elmer) was developed to quantitate APPΔC31 in the CHO-7W screening studies described herein. As shown in **Figures 2C–E**, biotinylated N-terminal APP antibody AF1168 (R&D) is bound to streptavidin-conjugated donor beads, and the Enzo anti-APPΔC31 antibody is used to coat acceptor beads. In the assay, 0.003 μL N-terminal anti-APP antibody/μL and 0.002 μL acceptor-bead-bound anti-APPΔC31 antibody/μL are added to samples in 1X HiBlock AlphaLISA buffer (Perkin-Elmer) on the assay plates. Plates are sealed and pulse centrifuged at 1000 rpm after each addition or mix step, shaken for 1 hr, and then incubated an additional hour without shaking in the dark. Streptavidin donor beads (PerkinElmer) are then added (1.5 μL, to give 30 μg/mL final), followed by 1 hr incubation with plate shaking. When streptavidin-donor beads are added to the reaction, they bind the biotinylated N-terminal APP antibody. When both antibodies bind with APPΔC31, they are in close enough proximity for excitation at 680 nm to result in the production of ^1^O_2_, interaction with the acceptor bead and emittance at 580 nm that is then detected using an Envision plate reader (Perkin-Elmer).

### ELISA to Detect APPΔC31

The APPΔC31 ELISA developed in partnership with Enzo Life Sciences (ADI-900-227) follows a standard sandwich ELISA design wherein a monoclonal N-terminal APP-specific antibody is used to coat the wells of the microtiter plate (capture antibody) and the anti-APPΔC31 cleavage site-specific polyclonal antibody serves as the detection antibody. An HRP-conjugated secondary antibody is then added and a colorimetric signal generated by enzymatic conversion of the tetramethylbenzidine (TMB) substrate. Protein levels were first determined using a colorimetric coomassie assay (Coomassie Plus, Thermo).

### Statin Induction of APPΔC31 Production

Since APPΔC31 is typically produced at very low levels and below the level of detection in the *in vitro* systems used here, it was necessary to induce this caspase cleavage in order to then knock it down. We previously reported the ability of statins, including simvastatin and cerivastatin, to stimulate the caspase cleavage of APP (Descamps et al., 2011). Here, we utilized this ability to discover APP C-terminal caspase cleavage inhibitors using CHO-7W cells. Cells were treated with activated simvastatin at 6 doses ranging from 100 nM to 10 μM, for 7.5 and 24 h, at which time APPΔC31 was measured in cell lysates using the AlphaLISA. Simvastatin was activated by opening the lactone ring before use in cell culture as previously described Dong et al. (2009); 8 mg simvastatin was dissolved in 0.2 mL of 100% ethanol (0.019 mM), with subsequent addition of 0.3 mL of 0.1 N NaOH and the solution was heated at 50°C for 2 h and then neutralized with HCl to pH 7.2. The resulting solution was brought to 1 mL final with distilled water, and aliquots were stored at -80°C.

In a separate experiment, CHO-7W cells were treated with: simvastatin, cerivastatin, and atorvastatin at 1, 5, and 10 μM, 5 μM simvastatin plus 30 μM pan-caspase inhibitor Q-VD-OPh (QVD, Sigma-Aldrich), and no statins, all for 24 h. APPΔC31 was determined in cell lysates by ELISA.

### High-Throughput Screening (HTS) for Inhibitors of the C-terminal Caspase Cleavage of APP

For HTS, CHO-7W cells were plated into 384-well Proxiplate SW plates (Perkin-Elmer) at 4K cells/well/10 μL in the growth media/conditions described above but also containing 1X Glutamax, 20 mM HEPES, 1X NEAA and 500 μg/ml G418. The following day, simvastatin at a final concentration of 10 μM and test compounds at 30 μM were added to assay plates via ECHO^®^ acoustic transfer (Labcyte). The next day, media was removed from the cells by “Flick and Slam” and centrifuging upside down. Cells were lysed in 3 μL of AlphaLISA buffer complemented with protease inhibitors on a plate shaker for 15 min.

The APPΔC31 AlphaLISA assay (**Figures 2C–E**) was run directly in the plate with the cell lysate as described above in “AlphaLISA to detect APPΔC31.” APPΔC31 levels were measured using the Envision plate reader and data were normalized to DMSO (100% APPΔC31 production) and Q-VD-OPh (MP Biomedicals) pan-caspase inhibitor at 30 μM (0%); these controls were present on each plate. In addition, some compounds of interest were further analyzed in a dose-response curve to determine IC_50_ values.

Compound libraries screened and confirmed included: (1) the US (1040 compounds) and International (240 compounds) Drug collections (Microsource); (2) the Enzo protease inhibitor library (60 compounds); and (3) a 903 compound natural products screen including a 502 compound Enzo natural products library, a 64 compound EMD inhibitors library, a 97 compound Enzo autophagy library, and a 240 compound GreenPharma natural products library.

### Validation of C-terminal APP Cleavage Inhibitors and Determination of Cell Viability

A subset of hits from our HTS was validated in a secondary assay. In addition, since the HTS hit spiperone has been reported to be a Wnt-signaling pathway inhibitor, we included in our testing another compound known to affect this pathway, thapsigargin, along with a spiperone analog 3′-fluorobenzylspiperone maleate (3′-fluorobenzylspiperone). Thapsigargin also acts as a sarco/endoplasmic reticulum calcium ATPase (SERCA) inhibitor. Therefore, another SERCA inhibitor, 2,5-Di-tert-butylhydroquinone, was also added to the testing. In this secondary assay, CHO-7W cells were cultured as described above and plated into 24-well dishes at ∼200K/well. The next day, cells were treated for 24 h in duplicate with 200 μL fresh media containing 10 μM cerivastatin (Sigma) to induce APPΔC31 production as well as select hits at 3, 10, and 30 μM, although the more potent thapsigargin was used at 0.033, 0.1, and 0.3 μM, and 2,5-Di-tert- butylhydroquinone at 1 and 10 μM. These hits were selected for their potency in primary screen, their structural features, their known activities, and availability of compound for additional testing. There were cerivastatin-only (negative control), + QVD-OPh (positive control), and for viability, vehicle-only (negative) controls. Cells were harvested by removing the media to a tube, adding 100 mL trypsin to detach cells then neutralizing with media. Cell viability (% live) was determined by trypan blue exclusion using BioRad’s TC-20 cell counter as it was important to ensure that any observed APPΔC31 inhibition was not due to inhibitor toxicity. Cells were centrifuged at 5000 rpm (2300 × *g*) for 5 min. Cell pellets were frozen until use. Forty μL cold RIPA buffer plus complete protease inhibitors (Roche) was added to each pellet and the pellets were reconstituted by pipetting up and down, incubating on ice for 30 min, vortexing, and centrifugation as above. Cell lysate supernatants were then assayed by APPΔC31 ELISA as described above. For most compounds, multiple/repeat studies were performed and two wells used for each condition. Statistical comparison was performed using one-way ANOVA for multiple comparisons, with a Dunnett *post hoc* test to compare each condition to the control. Further dose-response studies were performed on select compounds.

### *Ex vivo* Hippocampal Slice Cultures

Our lead APPΔC31 inhibitors were further tested *ex vivo* in I5 mouse organotypic hippocampal slice cultures in pilot experiments (**Supplementary Figure S4**). I5 mice express wt hAPP under the control of the platelet-derived growth factor promoter (Mucke et al., 2000). I5 mice originally purchased from Jackson labs (JAX B6.Cg-Tg(PDGFB-APP)5Lms/J) were bred and maintained in the Buck Institute vivarium facility, and used under the guidelines of an approved institutional IACUC protocol. P5–7 day mice were sacrificed using CO2 gas and cervical dislocation according to established IACUC protocols. Brains were removed, and under sterile conditions, hippocampi were dissected out on ice in Hybernate media + 1:400 Glutamax, 1:100 Pen/Strep and 1:50 B27 supplement (all Life Technologies). Hippocampi were laid parallel on a tissue chopper (McIlwain) in a small amount of media and sliced at 400 μm thick. Millicell-CM 6-well cell culture inserts (Millipore, 0.4 μm) were prehydrated by pipetting 750 μL culture media under each well and placing in a 35°C, 5% CO2 incubator for at least 30 min. Culture media: 100 mL MEM-HEPES, 50 mL HBSS, 50 mL HI horse serum, 1 mL 100x Pen/Strep (all Gibco), 1.3 g (6.5 mg/mL) glucose, and 2 mL 200 μM (2 mM) Glutamax, sterile filtered. Approximately equal portions of sliced hippocampi (4–5 pups/well) were placed onto inserts in minimal media (as stated above) and cultured for 5 days before treatment, changing the media under the insert every 2–3 days. After 5 days, treatment with vehicle (DMSO), cerivastatin (CS) at 30 μM, or CS plus 30 μM Q-VD-OPh, 10 μM spiperone, or 30 μM 3′-fluorobenzylspiperone for 72 h, with treatment being replaced under the insert each day. At the end of treatment, tissue was rinsed gently with PBS and harvested into a tube by gently scraping off the membrane with a scalpel using ∼100 μL/well cold RIPA buffer + protease inhibitors. Tissue was sonicated on ice for ∼5 s at “60” (Sonics Vibra Cell). Tissue sat on ice 30 min, was vortexed and centrifuged 5 min at 5000 RPM (2300 × *g*) before assaying the supernatant. Protein was quantitated for each sample using Coomassie Plus reagent (Thermo). An AMP’d Signal Amplification kit (Enzo) was used with the ELISA to detect APPΔC31 in these cultures as the signal was very low.

### Protein Concentration and sAPPα Levels

In an additional study (**Supplementary Figure S5A**), measurement of the α secretase fragment of full length (FL) APP – soluble APP alpha (sAPPα) – was performed using cell media by ELISA (IBL). Furthermore, in two studies (**Supplementary Figures S5B,C**), total protein levels in CHO-7W cell lysates were determined by Coomassie Plus protein assay (Pierce).

### Parallel Artificial Membrane Permeability Assay (PAMPA)

Several compounds emerged from testing as possible leads. Since the brain is the target tissue, brain-penetrance is a key feature of any potential new AD therapeutic. Immobilized artificial membrane (IAM) chromatography such as PAMPA (parallel artificial membrane permeability assay) is used to predict a compound’s passive diffusion through membranes and may predict blood-brain barrier (BBB) penetrance.

To minimize variability between PAMPA assays for different compounds, the well-characterized, reliable IAM.PC.DD2 Regis Technologies analytical columns were used. In these 10 cm × 4.6 mm columns, the phospholipid is bonded on 10 μm, 300 Å, spherical aminopropyl silica and end capped with C10 and C3 amides. It has been reported that, while column aging depends upon the column used as well as the test analyte, the intra-batch variability for IAM.PC.DD2 phases was small (Taillardat-Ertschinger et al., 2002). In addition, Ong et al. (1996) and others (Yoon et al., 2006; Shin et al., 2009) have shown that the IAM.PC.DD2 could be used as a rapid screening method for the prediction for drug absorption. In our hands, this PAMPA method has shown good correlation with brain-penetrance determined *in vivo* by pharmacokinetic (PK) analysis, and allows us to prioritize compounds for PK study.

Columns were connected to an Agilent high performance liquid chromatography system. Compounds (spiperone, pimozide, and 3′-fluorobenzylspiperone) were prepared at 10 μM in DMSO and diluted to 500 μM in 50:50 water:methanol. The mobile phase was a mixture of water and methanol and a gradient was used for the elution of the compounds. The detector was set at 250 and 280 nm. After a compound was eluted, the retention time and void volume times were obtained from the chromatogram.

The IAM capacity factor (KIAM) was calculated using the equation: $K_{IAM}=\frac{t_{r}-t_{0}}{t_{0}}$; where tr is the

retention time of a compounds and t0 is the void volume time of the column. It has been shown that the membrane permeability of a drug following passive diffusion is directly proportional to the K_IAM_ and inversely proportional to the molecular weight of a compound. To predict the likelihood a compound would be BBB penetrant, we used the correlation described by Yoon et al. (2006):

(I) If $\frac{K_{IAM}}{{MW}^{4}}{x10}^{10}$ > 0.85 the BBB penetration is predicted to be high
(II) If $\frac{K_{IAM}}{{MW}^{4}}{x10}^{10}$ < 0.85 the BBB penetration is predicted to be low

### *In vivo* Pharmacokinetic (PK) Analysis of Spiperone

As PAMPA is merely a predictive analysis of brain permeability, a pharmacokinetic (PK) study with spiperone was performed. Five adult (>3 months of age) male mice (C57B6, Jackson Labs) were injected subcutaneously (SQ) with 50 μL of spiperone at 5 mg/mL in 100% DMSO. Another 5 mice were injected intraperitoneally (IP) at the same dose. One mouse was euthanized from each dosing route group at 1, 2, 4, 6, and 8 h. This is done by ketamine/xylazine over- anesthesia followed by blood collection for plasma isolation by cardiac puncture, and transcardial perfusion of tissue with saline to remove residual blood from the brain. Both plasma and brain tissue were sent to Integrated Analytical Solutions (IAS, Berkeley, CA, USA) for compound level analysis using an LC-MS-MS method.

The animal testing was carried out in accordance with Public Health Service Policy on Humane Care and Use of Laboratory Animals, Assurance # A3196-01 and in the Buck Institute vivarium that has received AAALAC accreditation.

## Results

### Specificity of the APPΔC31 Antibody

Immunoblot analysis shows that the anti-APPΔC31 antibody is specific for caspase-cleaved APP, and does not react with full-length APP. As seen in **Figure 2A**, the band just below 100 kDa expressed after transfection of HEK 293T cells with *pcDNA3-APPΔC31*, but not for *pcDNA3-FL APP* is revealed by the antibody. In **Figure 2B**, again only APPΔC31 induced by treatment of CHO-7W cells with either staurosporine or simvastatin produced a band detectable with the antibody; FL APP present in lysates of untreated CHO-7W cells was not detected. In both cell types, two non-specific bands appeared (the lower is a triplet) and while the proteins in these bands have not been identified, they could be metabolites of APPΔC31 or other non-APP caspase-cleaved proteins with a similar antigenic site. It is important to note that in both the AlphaLISA and ELISA used for the screening and validation studies, a second N-terminal APP-specific antibody is used so that only APPΔC31 is detected and not shorter APP fragments.

Antibody specificity was also confirmed by analysis of purified MBP (maltose binding protein)-APPC125 (C-terminal 125 amino acids of APP including the β-, α-, γ-, and caspase-cleavage sites) fusion protein reactivity to anti-APPΔC31 as compared to anti-APP (6E10) by immunoblot where only anti-APP and not anti-APPΔC31 recognized this uncleaved fusion protein (**Supplementary Figure S1A**). In addition, overexpressed APPΔC31 and full-length APP_695_ from HEK 293T cell lysates were tested by APPΔC31 ELISA and showed similar results with the full-length APP lysate being almost undetectable with less than 1% cross-reactivity (**Supplementary Figure S1B**).

### Induction of C-terminal APP Cleavage Using Statins in CHO-7W Cells

**FIGURE 3:**
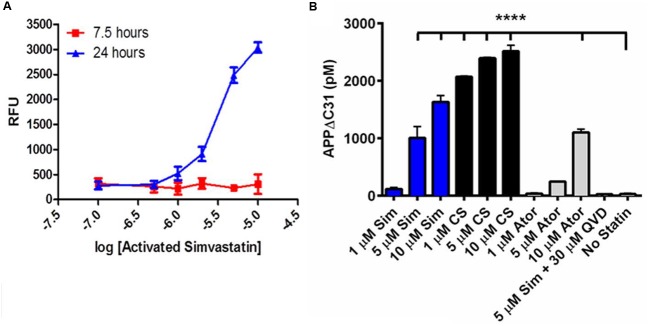
**Statins induce APPΔC31 production in a time- and dose-dependent manner.** **(A)** Activated simvastatin treatment of CHO-7W cells for 24 h, but not 7.5 h, leads to dose-dependent induction of APPΔC31 as determined by our APPΔC31 AlphaLISA assay; units are relative fluorescent units (RFU). Log^-5^ is 10 μM, log^-7^ is 100 nM. **(B)** Statin treatment of CHO-7W cells for 24 h leads to dose-dependent induction of APPΔC31 as determined by the APPΔC31 ELISA developed for these studies. Twenty-four hour simvastatin (Sim) increased APPΔC31 at 5 and 10 μM, and cerivastatin (CS) did so at 1, 5, and 10 μM. Atorvastatin (Ator) was much less effective. Pan-caspase inhibitor Q-VD-OPh prevented 5 μM simvastatin-induced APPΔC31 increases. Experiments were performed in duplicate and statistical analysis performed using ANOVA with Dunnett *post hoc* analysis to compare all compound effects to the “no statin” control (^∗∗∗∗^*p* < 0.0001).

As shown in **Figure 3A**, an initial time- and dose-response study showed simvastatin induction of APPΔC31 was both time and dose-dependent. Simvastatin increased APPΔC31 proportionally from 1 to 10 μM, and this was seen at 24, but not 7.5 h. Therefore, a 24-h treatment time was used for further studies. Simvastatin was then compared to cerivastatin and atorvastatin. Of the statins tested, cerivastatin was found to be the most effective at inducing the caspase cleavage of APP (**Figure 3B**). These results led us to use cerivastatin in the validation studies.

Induction of cerivastatin-induced APPΔC31 production was also seen by immunoblot. 3′-fluorobenzylspiperone decreased 10 μM CS-induced APPΔC31 at 30 μM here (boxed area). QVD at 30 μM eliminated the cerivastatin-induced APPΔC31 increase (**Supplementary Figure S2B**) and inhibited APPΔC31 production in a dose-dependent manner (**Supplementary Figure S2A**).

### Compound Library Screening for Inhibitors of the C-terminal Cleavage of APP

**FIGURE 4:**
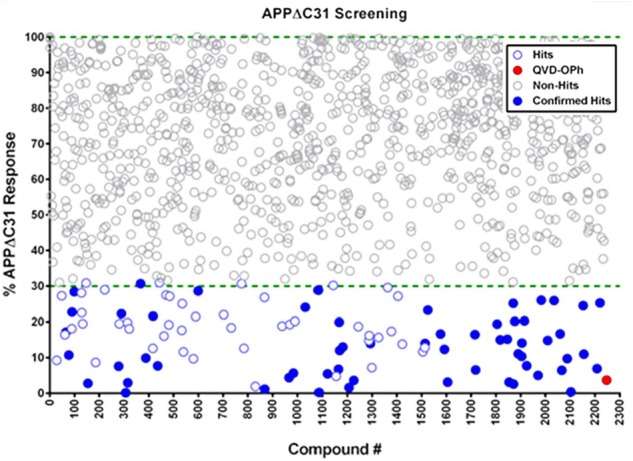
**Scatterplot of HTS hits.** Of 2243 compounds tested in HTS using the AlphaLISA, 91 hits – defined as compounds that inhibited simvastatin-induced APPΔC31 production by at least 70% – were found at a hit rate of 4%. The pan-caspase inhibitor Q-VD-OPh is represented by the red circle, and hits that were confirmed and went on to further evaluation are blue-filled circles (see **Supplementary Table S1**). Screening was performed in duplicate and the average coefficient of variation (CV) across all assays was 17 ± 4%.

A scatterplot (**Figure 4**) shows that a total of 91 hits out of 2243 compounds screened (4% hit rate) were found, using a hit criterion of at least 70% inhibition of APPΔC31 production. The red-filled circle represents the inhibition of APPΔC31 by the control pan-caspase inhibitor QVD-OPh, and hits initially selected for further evaluation that have undergone hit confirmation are represented by blue-filled circles. Screening was performed in duplicate and the average coefficient of variation (CV) across all assays was 17 ± 4%.

These hits comprised many different categories of compounds including histone acetyltransferase inhibitors, antibacterials, antifungals, cathepsin inhibitors, protease inhibitors, kinase inhibitors, mTOR inhibitors, natural products, protein synthesis inhibitors, phosphodiesterase inhibitors, Ca2^+^ channel blockers, Ca2^+^ signaling modulators, GPCR agonists and antagonists, and anti-inflammatory agents. Identities and APPΔC31 inhibition data for all confirmed hits from our HTS are shown in **Supplementary Table S1**. Potential therapeutic hits based on structure were cherry-picked for further validation and study.

### Validation of Inhibitors of the C-terminal Cleavage of APP

**FIGURE 5:**
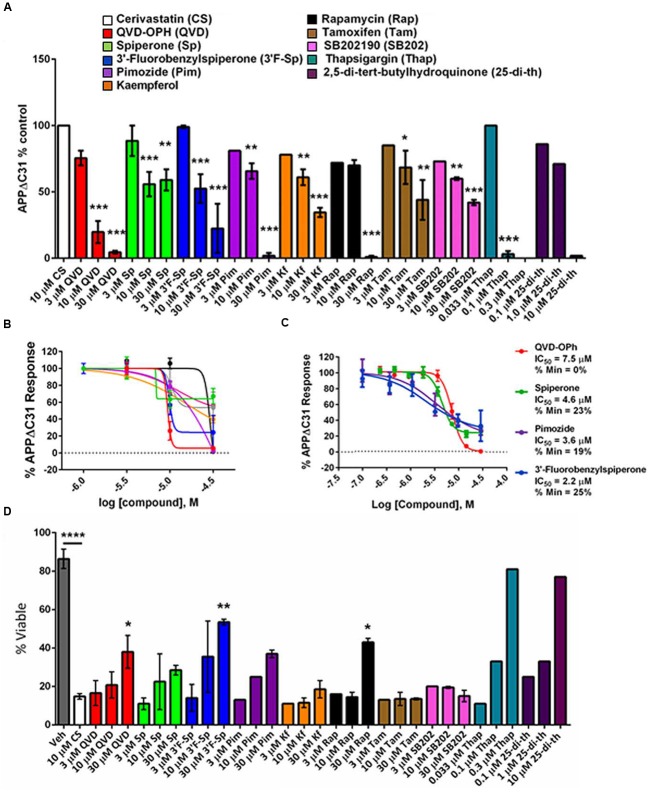
**Validation of select APPΔC31 inhibitor hits from screening.** **(A)** Hits revealed by HTS at 30 μM were retested in a larger well format at 3, 10, and 30 μM (unless otherwise specified) using cerivastatin to stimulate APPΔC31 production in CHO-7W cells. Q-VD-OPh (red) served as the control for caspase inhibition and the ELISA was used to determine APPΔC31 levels. Thapsigargin and 2,5-Di-tert-butylhydroquinone, while not identified in HTS, were added in validation studies due to their mechanism of action. *N* ≥ 2 for CS + QVD, Sp, or 3′F-Sp, and n = 1 – 2 for CS + Pim, Kf, Rap, Tam, SB202, Thap, or 25-di-th. Statistical analysis was performed using one-way ANOVA for multiple comparisons with Dunnett’s test to compare each treatment to CS at 10 μM alone (^∗^*p* ≤0.05, ^∗∗^*p* ≤0.005, ^∗∗∗^*p* ≤0.001). **(B,C)** In further validation studies, the dose-response was best for spiperone, 3′-fluorobenzylspiperone and pimozide. The ELISA was used for determination of APPΔC31 in **(B)**, and the AlphaLISA in **(C)**. **(D)** Increases in percentage of viable cells mirrored APPΔC31 reductions in a dose responsive manner. Untreated cells representing 100% viability are not shown while vehicle (DMSO) treated cells were ∼85% viable. Of the compounds identified in HTS, 3′-fluorobenzylspiperone elicited the greatest increase in cell survival; known SERCA inhibitors thapsigargin and 2,5-Di-tert- butylhydroquinone used at lower doses had the greatest effect on the rescue of cell viability. *N* ≥ 2 for veh, CS, QVD, Sp, and 3′F-Sp; *n* = 1 or 2 for Pim, Kf, Rap, Tam, SB202, Thap, and 25-di- th. Statistical analysis was performed as described above for **(A)** to compare compounds in combination with 10 μM CS to CS alone; in addition, CS alone was compared to the vehicle-only control.

Confirmed screening hits were validated for inhibition of APPΔC31 induction by ELISA using larger format cultures of CHO-7W cells, and several additional compounds were added in these secondary assays based on their known mechanisms of action. Specifically, since the hit spiperone is a known Wnt signaling inhibitor, another known inhibitor of Wnt signaling was analyzed as well, thapsigargin. Thapsigargin is also a SERCA inhibitor, and so another SERCA inhibitor, 2,5-Di-tert-butylhydroquinone was also analyzed. Dose-response analyses were performed on hits chosen for their potency and efficacy of APPΔC31 inhibition as well as some for structural features. Some of these compounds were run in 4- to 8-point dose-response curves as shown in **Figures 5B,C**.

Most of these compounds reduced cerivastatin-stimulated APPΔC31 in a dose-responsive manner after 24 h (**Figures 5A–C**) with Q-VD-OPh serving as the positive control. Thapsigargin (**Figure 5A**, **Supplementary Figure S3A**) proved to be a remarkably potent inhibitor of APPΔC31 production and another SERCA inhibitor, 2,5-Di-tert-butylhydroquinone, was also very effective. HTS hits spiperone, 3′-fluorobenzylspiperone maleate and pimozide were also potent inhibitors. The IC_50_ values (μM) of the lead compounds were: spiperone 4.6, pimozide 3.6, 3′-fluorobenzylspiperone maleate 2.2, and thapsigargin 0.077.

Cerivastatin induction of APPΔC31 and inhibition by Q-VD-OPh and 3′-fluorobenzylspiperone in a dose-dependent manner was also demonstrated by APPΔC31 immunoblots from CHO-7W cell lysates (**Supplementary Figure S2**).

Since APPΔC31 is generated by caspase cleavage and we found some MEK inhibitors in our HTS, select caspase and MEK inhibitors were also tested for inhibition of cerivastatin-induced APPΔC31 in CHO-7W cells for 24 h using dose-response curves determined by AlphaLISA (**Supplementary Figure S3B**). They included Caspase 6 Inhibitor I and II, Caspase Inhibitor X (inhibits caspases 3, 7, and 8), AZ10417808 (inhibits caspase 3) and Ivachtin (inhibits caspase 3). Although none of these caspase inhibitors were found to be very effective here, Caspase Inhibitor X (potency for various caspases: caspase 3 IC_50_ 4.3 μM; caspase 7 IC_50_ 6.2 μM; caspase 8 IC_50_ 2.7 μM) and Caspase 6 Inhibitor I at the highest concentration tested (30 μM) were somewhat effective. The broad-spectrum pan-caspase inhibitor Q-VD-OPh served as the positive control. The MEK1/2 (MAPK/ERK kinases) inhibitor U0126 reduced APPΔC31 ∼25% from 10 to 50 μM while the inactive isomer U0124 was ineffective. The MEK inhibitor SL327 was ineffective.

Lead APPΔC31 inhibitors were also tested in P5–7 day I5 wt mouse *ex vivo* organotypic hippocampal slice cultures (**Supplementary Figure S4**). These pilot studies were performed for lead compounds and it should be noted that the APPΔC31 signal was found to be low. Results within each experiment were protein normalized and are represented as % of cerivastatin control, which was set at 100% for each experiment, and thus there are no error bars for that treatment. Spiperone at 10 μM inhibited APPΔC31 production by ∼14% while 3′-fluorobenzylspiperone maleate at 30 μM inhibited it by ∼82%. Preliminary results suggest that these compounds are also efficacious *ex vivo* with, and some without, cerivastatin induction. Interestingly, pilot studies in primary I5 neuronal cultures treated with cerivastatin did not induce APPΔC31, additional investigation with neuronal cultures were not done as we found that the APPΔC31 could be induced by cerivastatin in slice cultures.

### Rescue of Cell Viability by Validated Hits

Cell viability was measured together with APPΔC31 since induction of APPΔC31 production (here stimulated by cerivastatin) increases cell death. It was also important to ensure that any observed APPΔC31 reduction was not due to compound toxicity. As seen in **Figure 5D**, increases in cell viability typically mirrored decreases in APPΔC31, confirming the toxic effect of the caspase cleavage of APP. Among hits from HTS, spiperone, pimozide, and rapamycin showed the greatest cell survival rescue effects at 30 μM. Of these, two compounds that were added to secondary screening due to their known mechanism(s) of action – thapsigargin and 2,5-Di- tert-butylhydroquinone – elicited the highest cell survival among compounds tested at the low concentrations. Complete APPΔC31 inhibition and viability data for all confirmed hits are shown in **Table 1**. Cell viability for hits tested represents rescue if greater than the level of cerivastatin treatment alone. DMSO represents maximal viability.

**Table 1:** Efficacy of lead APPΔC31 inhibitors.

Hit Name	Structure	MW	APPΔC31 Inh. (mean %)	Cell viability (mean %)	IC_50_
Spiperone	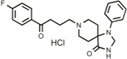	431	41	29	4.6 mM (A)

3′Fluorobenzylspiperone maleate	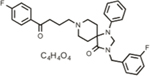	619	78	54	2.2 mM (A)

Pimozide	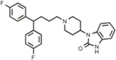	461	98	37	3.6 mM (A)

Thapsigargin	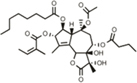	651	97	33 (@ 0.1 μM)	0.077 mM (E)

Kaempferol	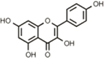	286	65	19	>10 mM (E)

Rapamycin	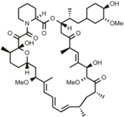	914	99	43	>10 mM (E)

Tamoxifen	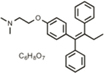	563	56	14	>10 mM (E)

SB202190	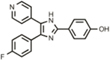	331	58	15	>10 mM (E)

SB216763	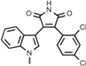	371	54	23	>10 mM (E)

QVD-OPh	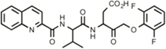	514	95	38	7.5 μM (E)

Cerivastatin	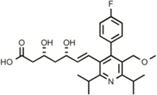	460	0	15 (@ l0 μM)	NA

DMSO	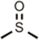	78	0	86	NA


*Compounds validated in secondary testing reveal their efficacy to both inhibit APPΔC31 and rescue CHO-7W cells from cerivastatin-induced cell death. Induction of APPΔC31 was accomplished with 10 μM cerivastatin and inhibition with 30 μM compound with up to 5 repeats.*

### Effects on Protein and sAPPα Levels

As shown in **Supplementary Figure S5A**, treatment of CHO-7W with QVD, spiperone and 3′fluorobenzylspiperone increased sAPPα in the presence 10 μM cerivastatin, did not significantly affect sAPPα indicating that these compounds do not affect APP expression. Furthermore, QVD alone at 30 μM and spiperone alone at 10 μM did not lower protein concentration in CHO-7W lysates as compared to vehicle as shown in **Supplementary Figure S5B**. In addition, and S5C QVD, spiperone and 3′fluorobenzylspiperone alone at 30, 10, and 10 μM, respectively, increased total protein concentration in the presence of 10 μM cerivastatin (**Supplementary Figure S5C**).

### PAMPA and PK

**FIGURE 6:**
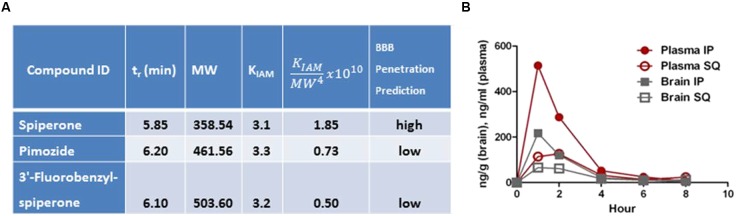
**Parallel artificial membrane permeability assay (PAMPA) and PK analysis.** **(A)** PAMPA analysis suggests that spiperone would have the highest likelihood of being brain-penetrant. **(B)** PK analysis indicated that after IP injection at 10 mg/kg, brain levels peaked (Cmax) at ∼205 ng/g at 1 h (*n* = 1 mouse per time point).

As shown in **Figure 6A**, of the three compounds tested in PAMPA, spiperone had the lowest tr and KIAM and the highest $\frac{K_{IAM}}{{MW}^{4}}{x10}^{10}$, and thus was predicted to be more brain-penetrant than pimozide or 3′-fluorobenzylspiperone maleate. This was not unexpected as spiperone also has the lowest MW of the three compounds. Pharmacokinetic analysis revealed spiperone did indeed enter the brain, peaking at 1 h for both the subcutaneous and intraperitoneal routes at ∼70 ng/g and ∼205 ng/g, respectively (**Figure 6B**). Plasma levels were higher at all time points and by both routes, with a brain:plasma ratio of ∼0.5.

## Discussion

Our laboratory and others (Lu et al., 2000; Banwait et al., 2008) have shown that the intracellular caspase cleavage of APP resulting in the production of APP-C31 is a destructive physiological process involved in AD. It has been shown that overexpression of APP-C31 in cortical neurons results in apoptotic cell death (McPhie et al., 2001), and that in a murine model of AD, prevention of APP-C31 generation by mutation of the D664 (of APP_695_) cleavage site reduced synaptic loss and improved synaptic transmission and spatial memory. Thus, identification of inhibitors of cleavage at this site would be instrumental in understanding the role of this cleavage in the disease process and represents a novel and potentially important new approach to therapeutic development in AD.

Statin-induced APPΔC31 (and thus APP-C31) increases could have therapeutic implications, especially for those at high risk for AD due to the expression of apolipoprotein 4 (ApoE4). Individuals expressing ApoE4 are at greater risk for both AD (Bertram et al., 2007; Mahley et al., 2007; Waring and Rosenberg, 2008) and cardiovascular disease (Mahley, 1988, 2016) and may receive statins as part of a program to lower their risk of the latter. Statins are 3-hydroxy-3-methylglutaryl-coenzyme A (HMG-CoA) reductase inhibitors used to inhibit cholesterol biosynthesis and in this way protect against vascular disease. While the exact mechanism is not known, memory loss associated with the use of statins has been reported (Strom et al., 2015) although there is concern around selection or detection bias in such reports. The risk of statin- induced memory impairment, however, should be considered for individuals at risk for AD. Cerivastatin was voluntarily withdrawn from the worldwide market in 2001 due to reports of fatal rhabdomyolysis (Bruno-Joyce et al., 2001). However, simvastatin, another lipophilic and highly brain-permeable statin effective in stimulating APPΔC31 production (albeit less so than cerivastatin), is still on the pharmaceutical market. Atorvastatin, also seen to increase APPΔC31 in our earlier studies (Descamps et al., 2011) as well as these studies, has a much weaker effect than do cerivastatin and simvastatin, and is hydrophilic with relatively low brain-permeability.

In contrast to what might be considered a risk associated with statin use, statins have been considered potential therapeutics for several CNS disorders, including ischemic stroke, AD, and multiple sclerosis (Ciurleo et al., 2014), as well as for cancer treatment (Werner et al., 2004; Cafforio et al., 2005; Minichsdorfer and Hohenegger, 2009). The effect of statins on the CNS includes effects that may be of benefit in AD prevention such as decreased Aβ production, increased Aβ clearance, and decreased reactive oxygen species (ROS) production as well as neuroinflammation. However, the effects of statins appear to be of less utility once AD is present (McFarland et al., 2014). Overall, there is thought to be a role of statins in reducing cholesterol in AD (Sun et al., 2015) but even this is not without controversy (Wood et al., 2014). As with all potential therapeutic approaches, the use of statins effectively may rest on appropriate diagnosis, timing, and individual patient cholesterol status.

Statins are also known to have anti-proliferative effects in certain tumor cell types (Baulch-Brown et al., 2007; Relja et al., 2010) and this effect is attributed in part to reduction of the isoprenylation of proteins involved in cell signal transduction such as Ras and RhoA (Cafforio et al., 2005). Cafforio et al. (2005) and others (Werner et al., 2004) have demonstrated that many statins, especially lipophilic ones such as cerivastatin, exert cytotoxic effects on tumor cells by directly promoting apoptosis through the intrinsic pathway involving mitochondria as opposed to the extrinsic death receptor pathway. A reduction in mitochondrial membrane potential and the cytosolic release of a second mitochondria-derived activator of caspases (Smac/DIABLO) was observed. They determined that this apoptotic pathway was caspase-dependent since caspases 9, 3 and 8 were activated; cerivastatin induced the maximum activation. They also showed that a caspase 9 inhibitor dramatically reduced apoptosis in MCC-2 cancer cells whereas a caspase 8 inhibitor did not. They therefore speculate that caspase 9 is the likely initiator whereas caspase 8 is subsequently transformed by caspase 3 into its active form. Caspase 8 activation by longer statin incubation times was confirmed in melanoma cells (Minichsdorfer and Hohenegger, 2009) although Werner et al. (2004) found that extrinsic pathways via death receptors through caspase 8 and calpain activation were not triggered by simvastatin and that deprivation of cholesterol precursors is essential for statin-induced apoptosis. Caspase 2 gene expression has been shown to be induced upon treatment with lovastatin in thyroid cancer cells (Zhong et al., 2003), and the caspase 7 gene has been identified as a novel statin-responsive gene (Gibot et al., 2009). In C6 glioma cells, statin (mevastatin and simvastatin) treatment led to the suppression of cell proliferation and induction of apoptosis (Yanae et al., 2011) where they measured an increase in caspase-3 activity through inhibition of geranylgeranyl diphosphate (GGPP) biosynthesis and found that these statins inhibit the activation of phosphorylated ERK 1/2 and Akt. Therefore, it is not surprising that our hit compounds not only inhibited the production of APPΔC31 and thus cytotoxic APP-C31, but they also rescued the CHO-7W cells from cerivastatin-induced cell death, over-riding the apparent statin-induced caspase activation.

As mentioned, analysis of the hits from our primary and secondary assays yielded many categories of potential targets such as histone acetyltransferase inhibitors, antibacterials, antifungals, cathepsin inhibitors, protease inhibitors, kinase inhibitors, mTOR inhibitors, natural products, protein synthesis inhibitors, phosphodiesterase inhibitors, Ca2^+^ channel blockers, Ca2^+^ signaling modulators, GPCR agonists and antagonists, and anti-inflammatory agents. While there may be a network of targets involved in this C-terminal APP cleavage, further work is needed to demonstrate their involvement such as knockout and overexpression experiments as well as validation using commercially available compounds or analogs directed against the specific targets. One could posit that the APPΔC31 reduction induced by the key compounds identified in this work could be due, in part, to a decrease in protein expression, or specifically expression of FL APP and this could lead to less FL APP interaction and reduced cell death (Park et al., 2009). However, our data on both total protein concentration and sAPPα levels from cells treated with these compounds alone or in combination with cerivastatin suggest that protein/APP expression lowering by these lead compounds is not a likely factor in their APPΔC31 reductions (**Supplementary Figure S5**). Furthermore, our key inhibitors reverse the cerivastatin-induced decrease in cell viability, and this rescue mirrored the reduction in APPΔC31 levels (**Figures 5A,D**). Nonetheless, in the future as these lead candidates or their analogs progress toward preclinical development additional mechanistic analysis will include determination of their effect on APP expression at both the RNA and protein levels.

At least two of our lead APPΔC31 inhibitors, spiperone and thapsigargin, are known to affect calcium mobilization induced by a Wnt ligand and to inhibit Wnt pathway activation (Lu and Carson, 2009; Thrasivoulou et al., 2013). The Wnt signaling pathway plays important roles in the regulation of cell proliferation, differentiation and apoptosis (Li et al., 2006). These findings suggest that regulation of intracellular calcium levels and thus the Wnt pathway could be playing a role in APPΔC31 and APP-C31 production.

It is not, however, a given that Wnt-signaling should be inhibited in AD. In fact, it may be contraindicated, at least in advanced AD. Inestrosa et al. (2012) reported a correlation between loss of Wnt signaling and Aβ toxicity as well as tau hyperphosphorylation, and that conversely, upregulation of the Wnt pathway prevented toxicity. In addition, Liu et al. indicated dysfunctional Wnt/β-catenin signaling affects BBB function and contributes to neurodegeneration in AD (Liu et al., 2014).

Since more APPΔC31 is found in early AD Braak staging, and not in late AD (Banwait et al., 2008), it may occur at a time when Wnt signaling compensation is underway in response to early dysfunction. In fact, it may be this compensation that leads, in part, to increased APPΔC31/APP-C31 production in early AD. Therefore, Wnt signaling inhibition may be appropriate at this time, but not later when Wnt signaling is downregulated. Further evidence that Wnt signaling should be supported in advanced AD comes from an extensive study of post-mortem AD brain wherein a wide range of alterations in components of the Wnt pathway was seen, likely resulting in dysfunction of the canonical Wnt pathway, particularly in the hippocampus and entorhinal cortex, two regions of brain severely compromised in AD (Riise et al., 2015). Not unexpectedly then, activation of the Wnt signaling pathway was shown to improve cognition in an AD mouse model (Skaper, 2014).

Our lead compound spiperone is an antipsychotic that acts as a potent dopamine D2, serotonin 5HT1A and 5HT2A antagonist and a sigma receptor ligand. The second lead compound, 3′-fluorobenzylspiperone, is a selective D2 ligand and also a strong APP C-terminal cleavage inhibitor. Anti-psychotics are frequently used in AD patients, and long-term use of antipsychotics has not been shown to either improve or worsen AD (Lopez et al., 2013), although it should be noted that spiperone itself is not commonly used. Spiperone is the only antipsychotic that shows an inhibitory effect on Wnt signaling (Lu and Carson, 2009); the other antipsychotic identified as a hit here, pimozide, also strongly inhibits APPΔC31 but does not inhibit Wnt signaling.

It is unclear whether these inhibitory compounds are acting directly or indirectly to inhibit the intracellular caspase cleavage of APP and what signaling mechanisms are involved, but it could be speculated that intracellular Ca2^+^ perturbations/flux could very well be involved since the SERCA inhibitor thapsigargin consistently produced such a dramatic effect (IC_50_ = 77 nM) including viability rescue. Our data also demonstrate that 2,5-Di-tert-butylhydroquinone – also a SERCA inhibitor – strongly inhibits APPΔC31 production. It is interesting to note that both have been reported to modulate intracellular calcium (Sakai and Teshima, 2001). Modulation of calcium levels may have a therapeutic role in AD since dysregulation of neuronal calcium levels in AD is well known and part of the ‘calcium hypothesis’ of AD (Khachaturian, 1987; Mattson, 1992, 1994). Dysregulation of intracellular Ca2^+^ in neurons and other cell types in the brain is strongly associated with chronic inflammation and AD (Tan et al., 2012; Brawek and Garaschuk, 2014) and calcium channel blockers may play a role in AD therapy (Tan et al., 2012). SERCA inhibition, however, operates at the level of the ER, and calcium channel blockers at the plasma membrane, although both have effects on cytoplasmic Ca2^+^. SERCA inhibitors and calcium channel blockers may have opposing effects, or SERCA inhibition achieves the desired effect *in vitro* and only under conditions of statin treatment, and may not be a desired mechanism *in vivo*. Further studies are needed to elucidate the role of each.

The chemical-genetics approach we used in this work allowed us to identify inhibitors of the intracellular caspase cleavage of APP and potential targets that warrant further evaluation for development of new AD therapeutics. We plan to evaluate the lead candidates identified in this discovery effort, such as spiperone, 3′-fluorobenzylspiperone and pimozide, in more than one murine model of AD. Here, compounds were identified and validated in cells and tissues expressing wt hAPP. We also plan to test our compounds in a mouse model carrying familial hAPP mutations. Based on these efficacy results, additional lead optimization efforts to identify effective APPΔC31 inhibitors would be conducted. Furthermore, we plan to perform HTS on the UCLA Molecular Screening Core compound library to identify additional inhibitors of APPΔC31 production.

## Author Contributions

VJ: Principal Investigator of the research described in this proposal. KP: Primary lab researcher for the western blots, ELISA and slice culture data described in this manuscript. She made major contributions to the preparation of the manuscript. DS: Performed all the HTS analysis described in this manuscript. PS: Performed the pharmacokinetic analysis (PK) and also made significant contributions to the preparation of the manuscript. JC: Performed the PAMPA assay and also was involved in optimization of the APPΔC31 AlphaLISA assay. BJ: Provided medicinal chemistry input. OD: Developed the APPΔC31 AlphaLISA assay. He also identified the effect of statins in increasing APP-C31 production. AM: Assisted KP with the testing of lead compounds in brain slice culture assay. MM: Developed at Enzo the APPΔC31 ELISA assay described in the manuscript in collaboration with KP and VJ. DB: Collaborator on the project. NC: Was the Co-PI of the research described in the manuscript. His lab primarily did all the HTS work described in the manuscript.

## Conflict of Interest Statement

The preparation of the APPΔC31 ELISA was done in collaboration with Enzo Life Sciences. However there was no financial relationship with Enzo Life Sciences. There is no royalties or other relationships relevant to the submitted work. The other authors declare that the research was conducted in the absence of any commercial or financial relationships that could be construed as a potential conflict of interest.

The reviewer DL declared a shared affiliation, though no other collaboration, with several authors (PS, JC, BJ, DB, and VJ) to the handling Editor, who ensured that the process nevertheless met the standards of a fair and objective review.

## Supplementary Material

FIGURE S1 **The APPΔC31 polyclonal antibody and ELISA are specific for APPΔC31 rather than full-length APP. (A)** Purified MBP (Maltose Binding Protein)-APPC125 (C-terminal 125 amino acids of APP including the β-, α-, γ- and caspase cleavage sites) fusion protein was the antigen electrophoresed, and anti-APP (6E10) or anti-APPΔC31 were the antibodies used to probe the immunoblot membranes. **(B)** For APPΔC31 ELISA validation, increasing doses of lysates from *pcDNA3-APPΔC31*-transfected or *pcDNA3-APP_695_*-transfected HEK 293T cells were compared.

FIGURE S2 **Cerivastatin induction of APPΔC31 and inhibition by 3′-fluorobenzylspiperone maleate and Q-VD-OPh as seen by immunoblot. (A)** CHO-7W cells were treated with 10 μM cerivastatin (CS) along with varying concentrations of Q-VD-OPh (QVD) for 24 h. Cell lysates were prepared and immunoprecipitated with anti-APP (6E10) overnight at 4°C and then incubation with Protein A/G beads (Santa Cruz Biotech). The resulting pellets were incubated, electrophoresed using Nupage 4–12% Bis-Tris gels (Life Technologies) and the 0.2 μm PVDF membrane probed with anti-APPΔC31. **(B)** CHO-7W cells were treated with increasing concentrations of CS or 10 μM CS together with increasing concentrations of 3′-fluorobenzylspiperone maleate (3′-F-Sp) or 30 μM QVD for 24 h. Lysates were prepared and membranes probed with anti-APPΔC31.

FIGURE S3 **Dose-response curves of APPΔC31 inhibition by thapsigargin and select caspase and MEK inhibitors (A)** CHO-7W cells were treated with 10 μM cerivastatin plus increasing doses of thapsigargin for 24 h. Lysates were prepared and APPΔC31 determined by ELISA. **(B)** CHO-7W cells were treated with 10 μM cerivastatin plus increasing doses of caspase and MEK inhibitors for 24 h. Lysates were prepared and APPΔC31 determined by AlphaLISA.

FIGURE S4 **Pilot testing of lead APPΔC31 inhibitors in *ex vivo* organotypic hippocampal slice cultures.** Hippocampal tissue from P5–7 day I5 wildtype APP mice was isolated, cultured and treated with 30 μM cerivastatin ± 30 μM Q-VD-OPh (QVD), 10 μM spiperone (Sp), or 30 μM 3′-fluorobenzylspiperone maleate (3′F-Sp) for 72 h. Tissue was harvested and lysates analyzed for APPΔC31 by ELISA. Lysates were protein normalized and expressed as a % of control.

FIGURE S5 **Effect of lead compounds on protein concentration and sAPPα. (A)** QVD, spiperone (Sp), and 3′fluorobenzylspiperone (3′F-Sp) at 10, 30, and 30 mM, respectively, in combination with 10 μM cerivastatin (CS) increased sAPPα determined in media from CHO-7W cultures treated for 24 h as compared to CS alone (*n* = 2). **(B)** Total protein from lysates of CHO-7W cells treated with either 30 μM QVD or 10 μM Sp alone for 24 h was similar to the vehicle control (*n* = 1). **(C)** Total protein from CHO-7W lysates treated with 10 μM CS alone or in combination with QVD, Sp, or 3′F-Sp at the concentrations shown increased (*n* = 1). Statistical analysis was performed using one-way ANOVA and Dunnett post-test for comparison to the 10 μM CS control (^∗∗^*p* < 0.001, ^∗∗∗^*p* < 0.0001).

TABLE S1 **Compounds identified as ‘Hits’ in HTS and confirmed.** Confirmed hits from the HTS are listed here along with their % APPΔC31 response.
