# Supplementary figures and images for: Screening for Small Molecule Inhibitors of Statin-Induced APP C-terminal Toxic Fragment Production
Source: Front Pharmacol. 2017 Feb 15;8:46. doi: 10.3389/fphar.2017.00046 (PMC5309220; doi:10.3389/fphar.2017.00046)

# Supplementary Files


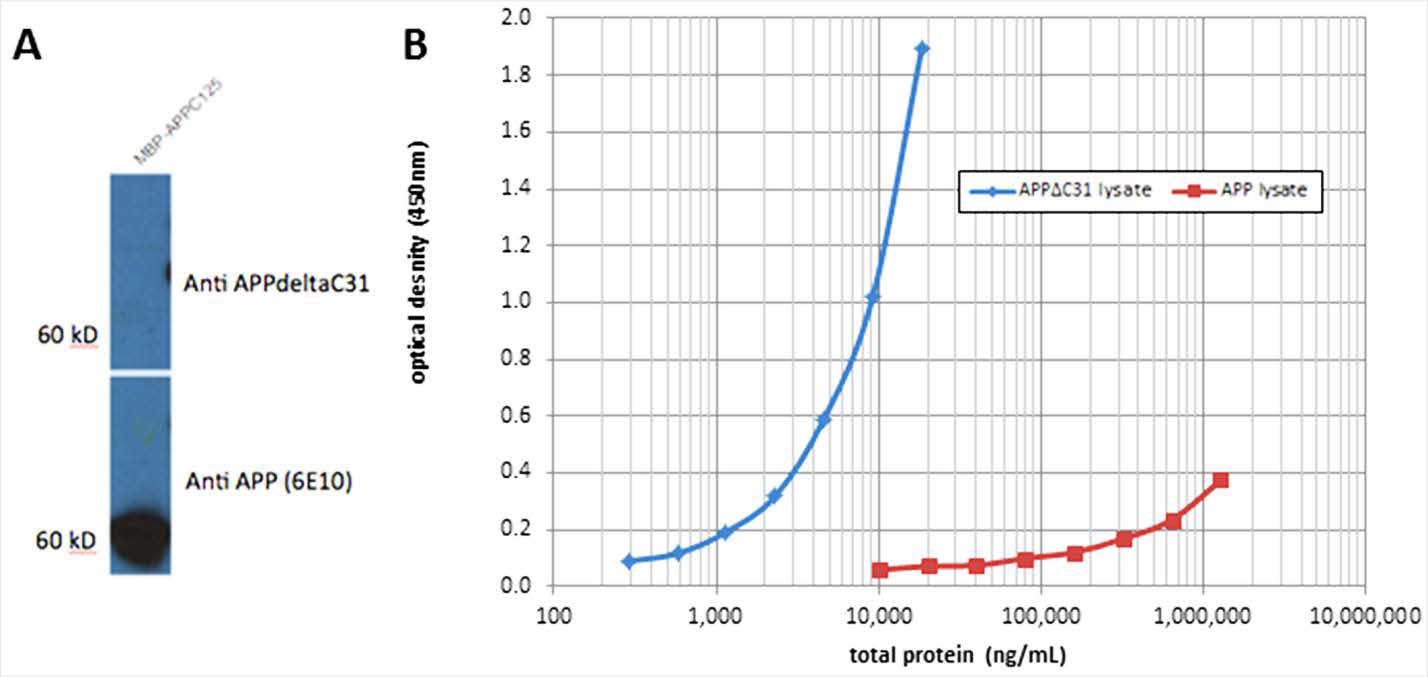
**Figure S1**


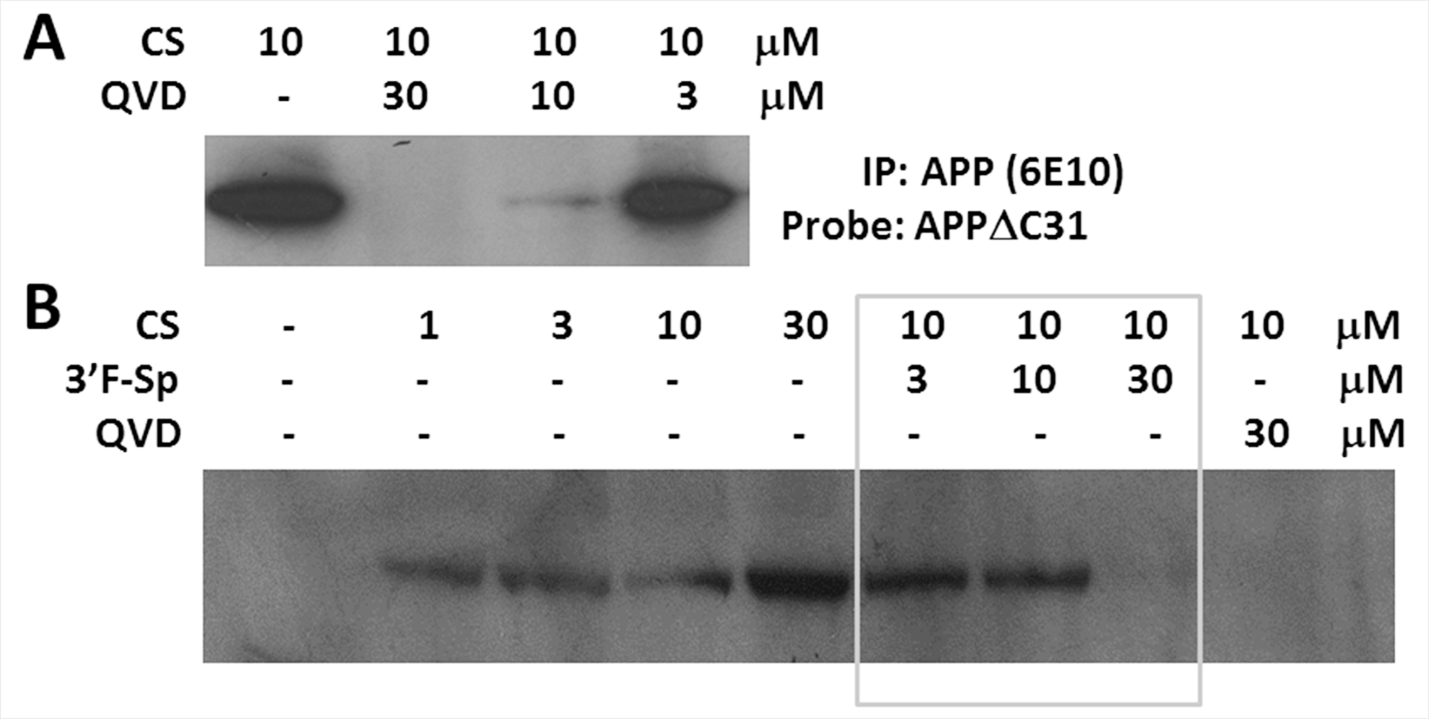
**Figure S2**


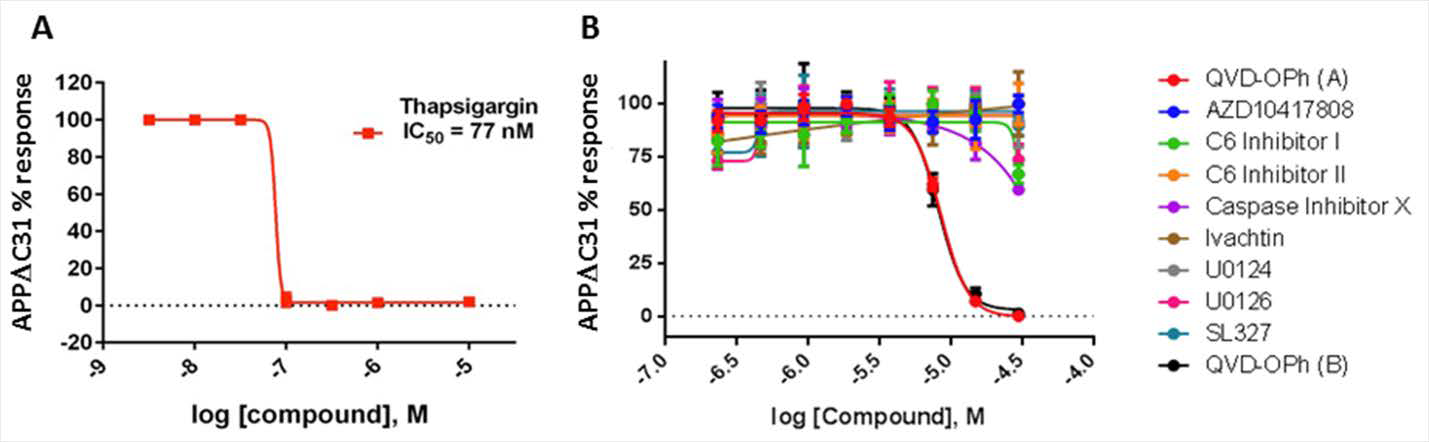
**Figure S3**


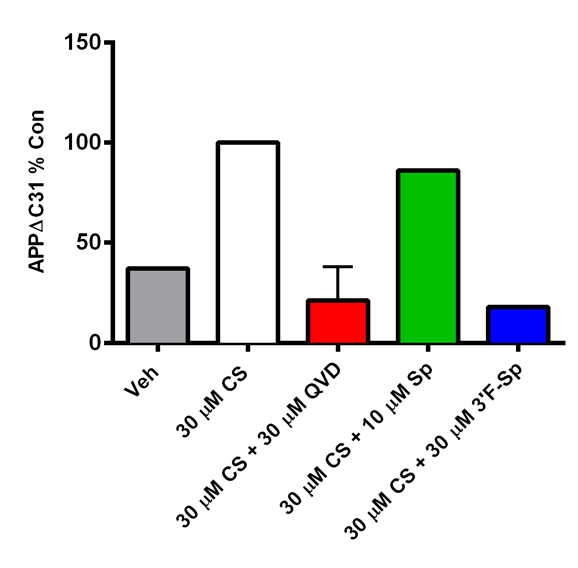
**Figure S4**

**Figure S5**


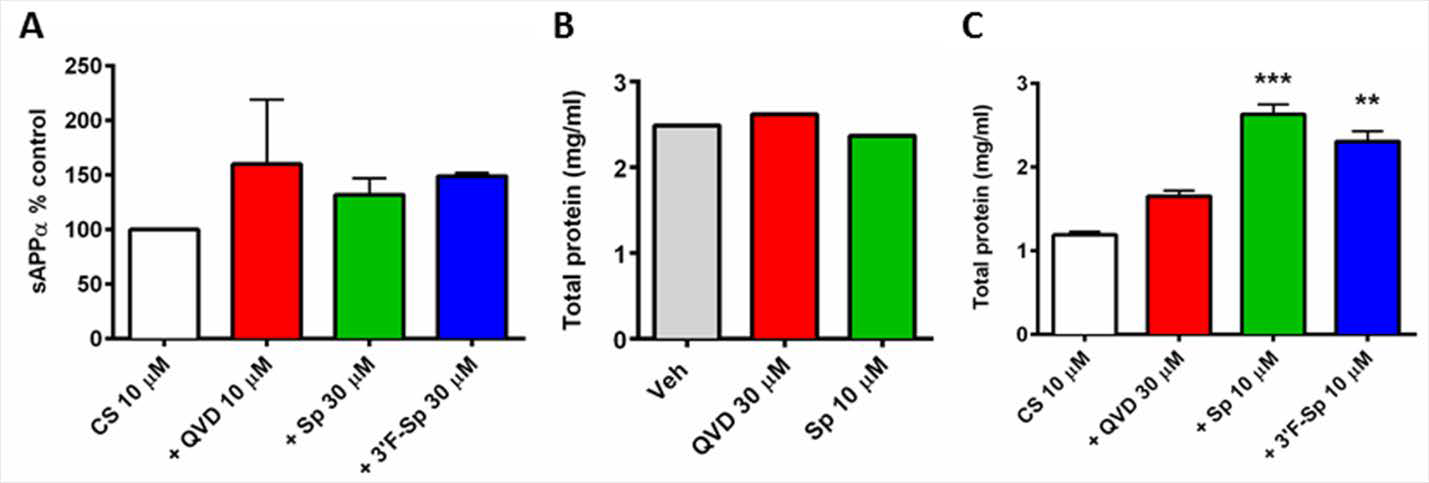

Supplement: FIGURE S1 — The APPΔC31 polyclonal antibody and ELISA are specific for APPΔC31 rather than full-length APP. (A) Purified MBP (Maltose Binding Protein)-APPC125 (C-terminal 125 amino acids of APP including the β-, α-, γ- and caspase cleavage sites) fusion protein was the antigen electrophoresed, and anti-APP (6E10) or anti-APPΔC31 were the antibodies used to probe the immunoblot membranes. (B) For APPΔC31 ELISA validation, increasing doses of lysates from pcDNA3-APPΔC31-transfected or pcDNA3-APP695-transfected HEK 293T cells were compared. [file Data_Sheet_1.docx]
