# Supplementary material for: Screening for Small Molecule Inhibitors of Statin-Induced APP C-terminal Toxic Fragment Production
Source: Front Pharmacol. 2017 Feb 15;8:46. doi: 10.3389/fphar.2017.00046 (PMC5309220; doi:10.3389/fphar.2017.00046)
Supplement: TABLE S1 — Compounds identified as ‘Hits’ in HTS and confirmed. Confirmed hits from the HTS are listed here along with their % APPΔC31 response. [file Table_1.pdf]

Structure	Common Name	Type / Target	% APPΔC31 Response	Structure	Common Name	Type / Target	% APPΔC31 Response
	1-(1h-indol-3-yl)-6,7-dimethoxy-1,2,3,4-tetrahydro-isoquinoline	natural product	19.4 ± 3.2		Isoxsuprine hydrochloride	Vasodilator	28.8 ± 0.2
	7-Oxozeaenol	Tak1 kinase inhibitor	3.8 ± 3.0		kinetin roboside	natural product	27.4 ± 7.8
	A-23187	calcium ionophore	4.4 ± 4.1		KN-93	CaMKII Inhibitor	3.3 ± 5.3
	ABC294640 HCl	SK2 inhibitor	26.6 ± 6.2		Lapidin	natural product	17.0 ± 4.8
	Akt Inhibitor IV	Akt Inhibitor	15.6 ± 7.6		Licochalcone-A	Bcl-2 / mTor inhibitor	2.5 ± 4.3
	Anisomycin	p38 MAPK activator	14.5 ± 13.5		Lycorine	natural product	1.0 ± 2.5
	Antimycin A1	natural product	11.0 ± 2.8		Macluroxanthone	natural product	25.8 ± 6.7
	artenimol	antimalarial / antiinflammatory	14.2 ± 8.5		MG-132	Proteasome inhibitor	13.4 ± 2.4
	Artesunate	natural product	16.5 ± 4.9		NapSul-Ile-Trp-CHO	Capthepsin L inhbitor	-0.3 ± 2.7
	Asarinin	natural product	7.0 ± 4.9		Narasin	natural product	1.4 ± 3.1
	BAY-43-9006	Raf-1 / B-Raf / Flt3 / KIT inhibitor	16.9 ± 6.3		Niclosamide	Antibiotic / Protonophore / mTor inhibitor	8.1 ± 2.6
	Bifonazole	Antifungal, calmodulin antagonist	12.9 ± 7.7		Nigericin sodium salt	stimulates mitochondrial ATPase activity	1.6 ± 3.8
	CA-074	Cathepsin B inhibitor	18.1 ± 2.9		Niguldipine	T-type calcium channel blocker	8.3 ± 5.8
	Camylofine dihydrochloride	Antimuscarinic	25.0 ± 9.2		Nimodipine	L-type calcium channel blocker	22.2 ± 8.4
	Cephadrine	antibacterial	4.0 ± 4.5		Nonactin	natural product	22.4 ± 6.5
	Cephaeline HBr	natural product	9.1 ± 9.7		Oligomycin	antifungal	7.1 ± 4.5
	Citreoviridin	natural product	20.6 ± 5.3		Parthenolide	antiinflammatory	14.2 ± 8.4
	Coumermycin A1	natural product	22.5 ± 8.9		Pimozide	D2 dopamine antagonist	4.2 ± 1.9
	Cyclohexamide	protein syntheis inhibitor	1.5 ± 4.8		Quinidine HCl	natural product	26.4 ± 16.7
	Cyclosporin A	Immunosuppressant	24.3 ± 4.5		Rotenone	Inhibitor of mitochondrial electron transport	12.0 ± 3.8
	D-cycloserine	NMDAR agonist	30.2 ± 5.9		SB 202190	p38 MAPK inhibitor	17.9 ± 4.1
	Degeulin	natural product	9.6 ± 3.6		SB 216763	GSK-3b inhibitor	0.9 ± 1.3
	Diacetoxyscirpenol	natural product	9.6 ± 4.0		Spiperone	Selective D2 dopamine receptor antagonist	30.0 ± 4.0
	Emetine dihydrochloride	RNA-protein translation inhibitor	3.9 ± 3.2		t2 toxin	natural product	10.7 ± 11.8
	Epicatechin	natural product	11.6 ± 6.0		Tamoxifen citrate	anti-estrogen and PKC inhibitor	25.3 ± 6.7
	Euphorbiasteroid	natural product	24.9 ± 4.0		Thapsigargin	SERCA inhibitor	6.2 ± 4.2
	Harringtonine	natural product	13.2 ± 13.5		Tryptanthrin	natural product	17.0 ± 6.3
	Hippeastrine	natural product	16.1 ± 4.3		Valinomycin	natural product	6.8 ± 2.5
	Histone acetyltransferase inhibitor II	p300 / Histone acetyltransferase inhibitor	26.7 ± 4.0		VEGFR Tyrosine Kinase Inhibitor	VEGFR Tyrosine Kinase Inhibitor	21.1 ± 4.1
	Homobutein	natural product	28.1 ± 4.9		Z-FA-FMK	Cathepsin B, L, S inhibitor	4.2 ± 2.8
